# Notes and News

**Published:** 1890-07-19

**Authors:** 


### NOTES AND NEWS.

COLLECTED AND COMMUNICATED.

There is another fasting man at the Aquarium, Jacques, who says he is going to fast for 42 days, and thus beat Succi. So far Jacques has got on well; he goes out more than Succi, and spends more time out of the wretched atmosphere of his room; he has not lost so much weight as Succi did so far. The scientific interest which attached to these exhibitions is dwindling now they are becoming mere public competitions for money. The public interest appears to be decreasing, and, doubtless, when a man has starved himself to death for the amusement of the many-headed beast, the many-headed beast will turn and formulate a law forbidding him to do it again. Would there were some law to hinder private starvation and unwilling starvation!

Probate of the will, dated August 16th, 1884, of the late Mr. Handel Cossham, of Weston Park, Bath, and Holly Lodge, in the parish of St. George, Gloucester, F.G.S., colliery proprietor, M.P. for East Bristol since 1885, who died at the National Liberal Club on April 23rd last, aged 65 years, has been granted to the acting executor. He leaves to the English Congregational Building Society £1,000 in colliery shares, to the National Temperance Society and Bristol and West of England Temperance Society £500 each, and to the Bath United Hospital £500. All these legacies may be paid in cash instead of shares at the option of the executors, and payment of the charitable bequests may be postponed. The testator devises and bequeaths all his real and personal estate, the personalty being of the value of £59,127 8s. 8d. in trust to accumulate the income during the life-time of Mrs. Cossham, and on her death, or within three years thereafter, to apply his residuary estate, so far as the law allows, to build on or near Kingswood Hill a hospital for the sick and injured of both sexes, "it being my earnest wish," the testator states in his will, "that I may be hereafter remembered by the sick and suffering as a friend who in death, as in life, has felt it his duty to try and lessen human suffering and increase human happiness." If land has not been given for the proposed hospital, the trustees are empowered to take or lease a site for it.

If any philanthropist wants to get what might be vulgarly called the worth of his money, he had better answer the appeal of Mr. Fox Bourne, secretary to the Aborigines Protection Society, for relief for the Hadendowas of Egypt. From a letter written on June 23rd by Dr. Harpur, medical missionary at Cairo, sent by the Church Missionary Society to inquire into the state of affairs at Suakin, Mr. Fox Bourne quotes as follows: "There are 2,000 outside in a continual state of starvation. There is a very large number of children among the distressed. Among 615 people I counted 287 small children apparently under eight years old, most of them nothing but skeletons, legs same thickness from foot to body, skin all hanging in folds." Mr. Bourne adds: "Twopence a day is the average cost of keeping one of these poor creatures alive; a guinea will maintain ten dozen."

Before our readers begin to disperse for the autumn holidays we wish them to give us a promise, or, rather, to make a vow unto themselves. It was very strongly brought before us last year that the adult patients of hospitals are more or less forgotten, while the children come in for most of the presents and pleasures. Of course, Christmas is essentially the festival of a Child and for children, but still the adult sick in the hospital wards have a large claim on our sympathy. It is so very hard to be absent from the home board—to miss the family roast beef and plum pudding. The readers of *Truth*, the Post Office employés, and many private persons, have now arranged that no sick child in the metropolis is left without a gift on Christmas Day. We do not expect to do as much as this for the grown-up patients, but, still, we should like to secure that the poorest and most suffering should receive some little present to help and comfort them. After all, the sick poor are few compared to the well who are in easy circumstances. If each of our readers—who number over 10,000 we know—would make us one garment by Christmas, or send us one gift, we would undertake to forward them to those hospitals where they were most needed. Such things as flannel petticoats, big linen aprons, woollen shawls, knitted waistcoats, mittens, comforters, and the old-fashioned cross-overs, would be very welcome. Some work we must have to occupy idle hours on the beach or wet days in lodgings; let it be work for the adult sick poor.

The Derby and Derbyshire Convalescent Home has celebrated its first anniversary in a convivial manner. Instead of a dull annual meeting the subscribers met at a lively luncheon on the lawn of the institution, and actually the weather was fine! Amongst the speakers was the Bishop of Derby, who was received with applause. He said he had great pleasure in moving the adoption of the report. He could fancy that some of them, as they heard it, might have thought that certain portions of it were somewhat disheartening. He wished to say, at first, that he thought it was anything but that. He knew, from experience, that the second report period of any institution was one of those which was most difficult to pass. It meant this: that an institution, when it began, began with a rush, and then arose difficulties which none of the committee, even with long foresight, had ever anticipated. Then came, sometimes, a downfall of hopes. Though there was, perhaps, something like that in the report, he was strongly of opinion that it showed absolutely sufficient buoyancy to ride safely over the second wave. It spoke of the applications for admission having been disappointingly small, but that time alone could cure. He had no doubt when the institution became better known throughout Derby and the county—when it became known to what a delightful place and to what invigorating air persons recovering from illness could come and recruit their health and strength—that difficulty would vanish. The meeting passed off exceedingly well.

The following vacancies are announced this week, the amount of the salary in each case being given after the name of the institution:—

**Resident Medical Officers.**

Dover Hospital and Dispensary, House Surgeon—£100, board, etc.

Birmingham General Hospital, two Assistant House Surgeons—board, etc.

Liverpool Northern Hospital, Assistant House Surgeon—£70, board, etc.

Liverpool Northern Hospital, Ambulance Surgeon—board, etc.

Lewes Infirmary, Resident Medical Officer—£100, rooms, etc.

Stockport Infirmary, Assistant House Surgeon—board, etc.

**Non-Resident Medical Officers.**

Hexham Sanitary Authority, Medical Officer of Health—£200.

Newcastle-on-Tyne Dispensary, Visiting Medical Assistant—£120.

Victoria University, Leeds, Demonstrator of Anatomy—£150.

Victoria University, Leeds, Demonstrator of Physiology.

New Hospital for Women, Clinical Assistant and Anæsthetist (Women).

Dr. Robertson issues the usual half-yearly statement of the affairs of the Devonshire Hospital and Buxton Bath Charity. During the half-year ending June 30th, 1,042 in-patients were admitted, and of this number 774 were discharged as "improved"; only 12 as "no better"; seven were discharged "at their own request"; one was found to be an "unfit case" for the hospital; three were found to be "infested with vermin"; three had to be sent away for "drunkenness"; the unusual number of eight "died" in the hospital; and 234 remained "on the books" at the end of the half-year. Twelve accidents were admitted during the half-year. Dr. Robertson himself is suffering from ill-health, and the committee express their sympathy with him.

Our hearty sympathy is with the employers and employés of the printing and kindred trades, who propose to establish a Claxton Seaside Convalescent Home and Sanatorium. The preliminary prospectus points out "(1) that the proposed institution would be our own, available for all connected with what, for brevity's sake, may be termed the printing and kindred trades; (2) that it would be managed by, and under the direct control of, representatives appointed or elected by the contributors; (3) That the home would be available in proportion to the amount subscribed, while the proposed constitution will amply safeguard all contributors in its management; (4) that the proposal cannot be considered a charity, but an union on a federal basis for the common philanthropic object of establishing and maintaining a seaside Convalescent Home for the kindred industries." Mr. R. W. Minter is the Chairman.

Many of the men employed in the caissons of the Forth Bridge were greatly affected by their work in an atmosphere of compressed air, and were in the habit of relieving the pain by spending their Saturday afternoons and Sundays in the air chamber. Mr. Muir, the engineer for Messrs. S. Pearson and Sons, of London, and who is now in charge of the Hudson Tunnel works, has, acting on the same idea, constructed a compressed air hospital for the men employed on the tunnel, amongst whom there have been several severe cases of "bends," although the air pressure is not particularly high, never, indeed, exceeding 30 lb: per square inch. The hospital (says *Engineering*) is a cylinder 18 ft. long by 6 ft. in diameter, constructed of steel plates ⅜ in. and ½ in. thick, and divided into two chambers by a transverse bulkhead. One of these chambers acts as an air-lock for the other, and both are fitted up with beds and everything necessary for the comfort of the patients. The air pressure is maintained by a pump, a constant supply of fresh air being secured by keeping a pet cock in the shell of the hospital open, through which the air continually leaks out. A safety valve is also supplied to prevent over-pressure should the pumps run away.

The annual report just issued by the Seamen's Hospital Society is drawn up in accordance with the scheme proposed in the *Hospital Annual*, which, if followed by all hospitals, would make the task of comparison of expenditure much more easy. Every penny is accounted for by this scheme, and the tables are easily understood. The report also possesses a map, showing the various branches of the work of the society; the "Dreadnought," at Greenwich, with 225 beds; the Branch Hospital, lately opened at the Albert Docks, and familiarly known as "Baby Dreadnought"; a Dispensary at the London Docks, and a Dispensary at Gravesend. The total expenditure last year was £11,632.

Reports are accumulating; that of Blandford Cottage Hospital is full and satisfactory, the matron being specially thanked for her thoughtful care. The London Skin Hospital treated 1,397 patients last year, and its President, the Marquis of Carmarthen, presided at the annual meeting on July 8th. Royston Cottage Hospital issues a very business-like little report, and the hon. secretary is to be congratulated on the statistical information. Islington Dispensary reports the disappearance of a deficit, but earnestly pleads for support for the Convalescent Fund. Bonchurch Convalescent Home had 77 patients last year, mostly from the Hants County Hospital; the Royal Hants Hospital itself treated 585 in-patients and 820 out-patients. The report is very full and satisfactory. Llandudno Cottage Hospital reports continued progress; a cot has been endowed during the past year.

Mr. Thomas Bryant has been elected President of the Royal College of Surgeons. Mr. Bryant's work as a man of science, as a practical surgeon, and as a teacher, well entitle him to this crowning honour. In Mr. Hutchinson the college had an illustrious head whose fame is in every part of the English-speaking world. Like Mr. Hutchinson, Mr. Bryant is not a mere surgeon, he is also a man of sterling sense, wide sympathies, and robust personal character. In these days men of Mr. Bryant's stamp are being more and more sought for to fill positions of public responsibility. The Royal College of Surgeons of England has always to bear in mind that it must present a face and a head to the public, as well as to the medical profession; and the men whom the public honour are those of strong natural intelligence, personal courage, and a stout resolution to choose the right course and to stand by it. Such a man is Mr. Bryant; and there is no doubt that during his term of office the College will have a President on whose judgment and experience it may safely rely, and of whom it may be justly proud.

Mr. F. Marsh, surgeon to the Queen's Hospital, gave evidence before the Hospital Reform Committee at Birmingham last week. Mr. Marsh, at the request of the committee, explained how the whole subject of hospital abuse arose. There was an informal meeting of members of the medical profession. A committee was appointed to consider the matter, and they elected a sub-committee to draw up a statement. That statement, in the shape of a printed report, was presented to a general meeting of medical men living within twenty-five miles of Birmingham. About 500 were summoned to the meeting, and 90 attended. They were unanimous in adopting it, and all the resolutions were carried *nem. con.* The meeting, he thought, represented fairly all classes of the profession. There was a general feeling amongst hospital officers that they had a great many trivial cases to attend to, and in conversation with practitioners one had complaints of the monetary abuse. In conclusion, Mr. Marsh said he thought the best reform would be in the formation of a provident system to which the trivial cases might be referred. The object would be to place medical treatment without the intervention of charity within the reach of more people. The subscribers would repay weekly, or at less frequent intervals if they desired, on a graduated scale, decided by income or house rent, and adjusted to the domestic demands upon the patient. The medical attendance would be by medical men in the district who signified their desire to join the service, and the payment according to work done.

